# Preference of urban and rural older people in Shandong Province for long-term care insurance: based on discrete choice experiment

**DOI:** 10.3389/fpubh.2024.1445273

**Published:** 2024-10-15

**Authors:** Wenxue Jin, Junlei Wang, Xiaoqian Hu

**Affiliations:** School of Politics and Public Administration, Qingdao University, Qingdao, China

**Keywords:** long-term care insurance, older people, preference, discrete choice experiment, China

## Abstract

**Background:**

Severe population ageing and weak long-term care support systems has spurred China’s pilot program for long-term care insurance (LTCI). This study aimed to provide references for optimizing long-term care insurance policies in Shandong Province by measuring the preferences of urban and rural older people for LTCI.

**Methods:**

Based on the discrete choice experiment, a questionnaire survey was conducted on urban and rural older people from Shandong Province. A mixed logit model was used for data analysis and the relative importance of attributes, willingness to pay, and preference heterogeneity based on residence type, number of children, chronic conditions, gender, education level and financial situation were further estimated.

**Results:**

The results showed that individual premium, reimbursement rate and whose provision of home-based care can be reimbursed had a significant effect on the LTCI preference of urban and rural older people in Shandong Province. Benefit package and government subsidy lost statistical significance in full sample but played a role in certain subgroups. There were also differences in preferences for individual premium among different groups of older people.

**Conclusion:**

Optimizing the policy design of long-term care insurance based on the actual needs of the older adults can help increase the utility of them and promote the smooth implementation of long-term care insurance.

## 1 Introduction

Population ageing has become a global challenge today (1). Data released by China National Bureau of Statistics showed that the proportion of older people aged 65 and above in China was as high as 14.9 percent by the end of 2022. According to the United Nations’ classification standards for aging, this means that China has already entered the stage of moderate aging. Currently, China’s population aging is continuing to accelerate (2). With the deepening of the degree of aging and the increase in the number of senior older people, the scale of disabled older people will further expand (3), making it a challenge for Chinese society to meet the huge demand for long-term care of the disabled older people.

Traditionally, the long-term care needs of older people in China were basically met within the family (4–6). However, with the miniaturization of family structure and the increase in female labor force participation rates, the resources available for family care have decreased, and the opportunity costs for family members to provide care have increased (7, 8). Meanwhile, specialized care services are often so costly that families and individuals cannot afford it (7). Caring for the disabled older adult places a heavy physical, psychological and financial burden on family members (3). The problem of long-term care can no longer be solved at the individual and family levels. Therefore, it is necessary to establish a socialized care guarantee mechanism to alleviate the burden on family caregivers (9).

Long-term care insurance (LTCI) is an effective policy tool to deal with disability risks (10), which can be categorized into public long-term care insurance and private long-term care insurance (1). The Netherlands passed the Exceptional Medical Expenses Act in 1967, which was formally implemented in 1968, becoming the first country in the world to establish a mandatory public LTCI scheme (11). Subsequently, Germany and Japan passed LTCI legislation in 1994 and 1997 respectively, establishing public LTCI (12). The United States of America developed private LTCI in the 1970s, and in addition to America, the private LTCI market in France is relatively well developed (13). In order to actively cope with the aging of the population and solve the problem of “long-term care service deficit” formed under the double squeeze of the increasing demand for long-term care services of older people and the weakening of the family care function, China carried out a pilot scheme of public long-term care insurance in 2016. By the end of 2022, a total of 169,902,000 people in 49 pilot areas had participated in the insurance scheme, and 1,208,000 people were enjoying the benefits of long-term care insurance (14). The system design of long-term care insurance followed the path of medical insurance and was divided into employee long-term care insurance and resident long-term care insurance, with resident long-term care insurance covering both urban and rural residents. Shandong Province, as one of the key pilot provinces for long-term care insurance, has fully implemented employee long-term care insurance in 16 prefecture-level cities, becoming the first province in the country to achieve full coverage of employee long-term care insurance, and has set a working target of achieving full coverage of resident long-term care insurance by 2025.Broad public support is an important prerequisite for the success of a public policy (15). Older people are the group most closely related to long-term care insurance. Therefore, it is necessary to understand the real preferences of older people for long-term care insurance, and design a long-term care insurance that meets their needs to increase the participation rate of long-term care insurance and ultimately contribute to the sustainable development of LTCI.

Discrete choice experiment (DCE), as an econometric method to measure preferences, is increasingly used in the health field. However, there are relatively few studies on long-term care insurance preferences using DCE. Brau et al. (16) firstly used DCE to study the LTCI choice preferences of the population in Emilia-Romagna region of Italy. Subsequently, Thailand (17), the Netherlands (18), the United States (19) and other countries applied DCE to measure preferences for long-term care insurance separately. In addition, some other scholars have studied the preference and willingness to pay for long-term care services (facilities) (20–22). Most of these studies have targeted service recipients, and individual article has measured the preferences of long-term care service providers (23).

In terms of research in China, He et al. (24) studied the preferences of middle-aged people in Hong Kong for private LTCI. Ma et al. (25) conducted DCE studies targeting public LTCI among middle-aged and older adult residents and Wang et al. (26) used DCE to measure the preference for long-term care insurance among people aged 20–75 in Liaoning Province. There is currently no research specifically targeting the preferences of urban and rural older people for long-term care insurance. This paper focused on older people in urban and rural areas of Shandong Province, and used discrete choice experiment to simulate the attribute level combinations of long-term care insurance to measure the stated preferences for long-term care insurance attributes among urban and rural older people in Shandong Province, so as to provide references for the improvement of long-term care insurance policies, increase the attractiveness of the long-term care insurance, expand the coverage of LTCI, and promote the sustainability of long-term care insurance.

## 2 Research design

In order to understand the true preferences of older people for long-term care insurance, this study used a discrete choice experiment to investigate. Discrete choice experiment, originating from random utility theory, is an econometric technique used to measure target group’s preference for a particular characteristic of a good or service (27). It simulates the actual choice situation by providing products with different combinations of attributes to identify the respondents’ real choice intention.

### 2.1 Development of DCE attributes and attribute levels

The development of attributes and levels is fundamental to the implementation of discrete choice experiment, and designing the appropriate attributes and levels largely determines the effectiveness of discrete choice experiment (28). Methods currently used to develop DCE attributes and levels mainly include literature review, semi-structured interviews, focus group discussions, theoretical argumentation, etc. (29, 30). In this paper, we first identified eight LTCI attributes through literature review: individual premium, care facilities, caregivers, government subsidy, benefit package, reimbursement rate, whose provision of home-based care can be reimbursed, elimination period (16–19, 25, 26, 31). Second, elimination period was excluded through LTCI policy analysis and focus group discussions because most LTCI pilot cities in China did not cover elimination period in their policies. Finally, we conducted in-depth interviews with one insurance executive, one policy maker and six academic experts and excluded two attributes: care facilities and caregivers, because they were considered features of long-term care services rather than insurance. Attribute levels were determined based on policies being applied to LTCI pilots in Shandong Province and were adjusted according to expert advice. For example, government subsidy for long-term care insurance in Shandong Province ranges from 0RMB-40RMB. Therefore, we set the range of values for the government subsidy as 0RMB-40RMB and took 20RMB as the middle value. Finally, four attributes were assigned 3 levels and one attribute was assigned 2 levels. The appropriate number of levels is 2–4 (32), and our setting fell within this range. Table 1 presents the five attributes and their levels included in the final design.

**Table 1:** The attributes and levels of LTCI scheme in DCE.

Attributes	Description	Levels
Individual premium	The amount of money insured pays every year	20RMB/year, 70RMB/year, 120RMB/year
Government subsidy	Government contributions to the LTCI fund every year	0RMB/year, 20RMB/year, 40RMB/year
Benefit package	Services that insured can receive from LTCI	Medical care + basic life care, Medical care + basic life care + rehabilitation training, Medical care + basic life care + rental of assistive devices
Reimbursement rate	Proportion of long-term care expenditure borne by the LTCI fund	65,75,85%
Whose provision of home-based care can be reimbursed	Whether to restrict the care providers in LTCI scheme	Professional caregivers only, Professional caregivers and family members, relatives and neighbors et al.

### 2.2 Experimental design and questionnaire development

After determining the attributes and levels of long-term care insurance, the next step is to conduct an experimental design, i.e., formulating specific combinations of attributes and levels that respondents evaluate in choice questions. The experimental design in general can be divided into two methods: full factorial design and partial factorial design (33). In order to improve the efficiency of the experiment, this paper adopted the method of partial factorial design, and created an experimental design containing 18 choice sets with two options in each choice set through the % ChoicEff macro of SAS 9.4 software. The D-efficiency value of the SAS experimental design is 13.7871, and the D-error value is 0.0725.Since the research object of this paper is older people, in order to reduce the cognitive load of the subjects, this study divided these 18 choice sets into three blocks, i.e., each respondent was actually faced with six choice scenarios. Example of a choice set is shown in Table 2. In addition to the choice set questions, the questionnaire also contained demographic information such as age, gender, marital status, and years of education, etc.

**Table 2:** Example of a choice set.

Attributes	Long-term care insurance A	Long-term care insurance B
Individual premium	120RMB/year	70RMB/year
Government subsidy	40RMB/year	20RMB/year
Benefit package	Medical care + basic life care + rehabilitation training	Medical care + basic life care + rental of assistive devices
Reimbursement rate	65%	85%
Whose provision of home-based care can be reimbursed	Professional caregivers and family members, relatives and neighbors et al.	Professional caregivers only
Your choice (tick one)	□	□

### 2.3 Sampling and data collection

This study took urban and rural older people in Shandong Province as the survey object, and the inclusion criteria of the survey object were: age of 60 years and above; permanent population in the survey area; can communicate normally and have no mental disorder. The survey was conducted using a multi-stage stratified sampling method, in which Shandong Province was first divided into economically developed regions, economically average regions, and economically underdeveloped regions according to GDP, and then two urban communities and two rural villages were randomly selected in each region, and finally older people meeting the inclusion criteria were randomly selected in each urban community or rural village. Community and village committee staff assisted us in inviting older people meeting the inclusion criteria to the conference room to finish the questionnaire surveys on the demand for long term care insurance.

According to the rule of thumb (34): *n* > 500c/(t × a), the minimum sample size required for a discrete choice experiment can be calculated. In the formula, n represents the minimum sample size, t represents the number of choice sets, a represents the number of alternatives in each choice set, and c represents the maximum value of the number of attribute levels. In this study, t = 6, a = 2, and c = 3, which led to a minimum sample size of 125 for this study. In order to improve the accuracy of the estimation and to keep the sampling error as small as possible, this study expanded the sample size to 360, which means that 30 older people were randomly selected from each urban community or rural village to conduct a survey on the need for long-term care insurance. Considering the complexity of the questionnaire questions, the questionnaire survey was conducted in the form of one-on-one interviews, and 360 questionnaires were distributed. After data cleaning, we deleted 15 samples with missing values.345 valid questionnaires were obtained, and the recovery rate of valid questionnaires was 95.83%. This study was approved by the Medical Ethics Committee of the School of Public Health, Zhejiang University (Approval number:ZGL202308-2).

### 2.4 Statistical analysis

In this paper, Stata16.0 software was used to construct a mixed logit model to analyse the choice preferences of older people for long-term care insurance. We set individual premium as a fixed parameter and other attributes as random parameters that follow a normal distribution. The utility that individual i derives from alternative j is given by:


$$\begin{array}{l} U_{ij=}\beta_{0}+\beta_{1}\mathrm{premium}+\left(\beta_{2}+\omega_{2i}\right)\;{\mathrm{subsidy}}_{20}\\ \;+\left(\beta_{3}+\omega_{3i}\right)\;{\mathrm{subsidy}}_{40}+\left(\beta_{4+}\omega_{4i}\right)\;{\mathrm{package}}_{1}\\ \;+\left(\beta_{5}+\omega_{5i}\right)\;{\mathrm{package}}_{2}+\left(\beta_{6}+\omega_{6i}\right)\;{\mathrm{proportion}}_{75}\\ \;+\left(\beta_{7}+\omega_{7i}\right)\;{\mathrm{proportion}}_{85}+\left(\beta_{8}+\omega_{8i}\right)\;\mathrm{family}+\varepsilon_{ij}\text{.} \end{array}$$

Here premium is individual premium; subsidy_20_ and subsidy_40_, respectively, represent government subsidy of 20 RMB/year and 40 RMB/year; package_1_ and package_2_, respectively, represent the welfare package of “medical care + basic living care” and “medical care + basic living care + assistive device rental”;proportion_75_ and proportion_85_ represent reimbursement ratio of 75 and 85%, respectively; family represents professional caregivers and family members, relatives, and neighbors’ provision of home-based care all can be reimbursed. *β*_0_ is the constant term, *β*_1_-*β*_8_ represent the mean of each attribute coefficient, *ω*_2i_-*ω*_8i_ represent the standard deviation of the attribute coefficients, and *ε_ij_* is the error term.

On the basis of the mixed logit model, this paper calculated the ratio of the regression coefficient of other LTCI attribute levels to the regression coefficient of individual premium to obtain the price that older people were willing to pay for each attribute level of LTCI (Willingness To Pay, WTP). Finally, subgroup analyses by residence type, number of children, chronic conditions, gender, education level and financial situation were conducted to understand preference heterogeneity. Differences were considered statistically significant at *p* < 0.05.

## 3 Results

### 3.1 Descriptive statistics

The survey respondents were predominantly older people who were 60–79 years old, with fewer senior older people. The ratio of men to women was basically equal, with women slightly outnumbering men.51.30% of the respondents were from rural areas and 48.70% were from urban areas. Most of the respondents were poorly educated, in marriage, had two or more children and with chronic diseases. The questionnaire used the question “What do you think your family’s living standard is in the local area?” to measure the financial status of the respondents, and the survey found that the older people who were living in difficulty and those who were living in affluence were in the minority, and the majority of the older people (57.68%) thought that their lives were in the general level in the local area (Table 3).

**Table 3:** Basic characteristics of survey respondents.

Variable		Frequency (person)	Proportion (%)
Age	60–69	182	52.75%
	70–79	136	39.42%
	≥80	27	7.83%
Gender	Male	164	47.54%
	Female	181	52.46%
Residence type	Rural	177	51.30%
	Urban	168	48.70%
Education level	Uneducated (years of education≤6)	225	65.22%
	Educated (years of education≥7)	120	34.78%
Marital status	In marriage	264	76.52%
	Not in marriage (including unmarried, divorced and widowed)	81	23.48%
Number of children	≤1	69	20.00%
	≥2	276	80.00%
Chronic conditions	Without chronic diseases	116	33.62%
	With chronic diseases	229	66.38%
Living standards	Difficult	31	8.99%
	Common	199	57.68%
	Affluent	115	33.33%

### 3.2 Preference for LTCI

This paper discussed the results of preference analysis mainly based on the mixed logit model. According to the main-effect model of mixed logit, three attributes, namely individual premium, reimbursement rate and whose provision of home-based care can be reimbursed, had a significant effect on the long-term care insurance choice preference of urban and rural older people in Shandong Province (*p* < 0.05), while government subsidy and benefit package lost statistical significance in mixed logit model, indicating that these two features were not among the key issues of consideration when older people were making their decisions.

In terms of individual premium, the regression coefficient was negative, indicating that an increase in individual premium will gradually reduce the utility of the older people. Compared with 65% reimbursement rate, 75 and 85% reimbursement rates will bring more utility to the older people. Taking “only professional caregivers’ provision of home-based care can be reimbursed” as the reference level, older people preferred long-term care insurance where professional caregivers and family members, relatives, and neighbors’ provision of home-based care all can be reimbursed. The calculation of willingness to pay showed that older people were willing to pay an additional 78.582RMB and 128.429RMB respectively, thereby increasing the reimbursement rate from 65 to 75 and 85%, and were willing to pay an additional 91.091RMB for long-term care insurance that reimburses homed-based care provided by both professional caregivers and family members, relatives and neighbors (see Table 4).

**Table 4:** Long-term care insurance preference results based on a mixed logit main-effect model.

Attributes and Levels	Mean	SD	WTP/RMB
Constant term	−0.424^***^	–	–
Individual premium (included in real terms)	−0.009 ^***^	–	–
Government subsidy			
0RMB/year	Reference	–	–
20RMB/year	0.159	−0.004	18.445
40 RMB/year	0.038	0.021	4.414
Benefit package			
Medical care + basic life care + rehabilitation training	Reference	–	–
Medical care + basic life care + rental of assistive devices	−0.153	0.001	−17.855
Medical care + basic life care	0.171	0.513^***^	19.867
Reimbursement rate			
65%	Reference	–	–
75%	0.675 ^***^	0.455^*^	78.582
85%	1.104^***^	0.903^***^	128.429
Whose provision of home-based care can be reimbursed			
Professional caregivers only	Reference	–	–
Professional caregivers and family members, etc.	0.783 ^***^	1.351^***^	91.091
Log likelihood	−1233.274		
Sample size	345		
Observed value	4,140		

SD, standard deviation. ***p < 0.001, **p < 0.01, *p < 0.05.

### 3.3 Relative importance of attributes

**Figure 1:**
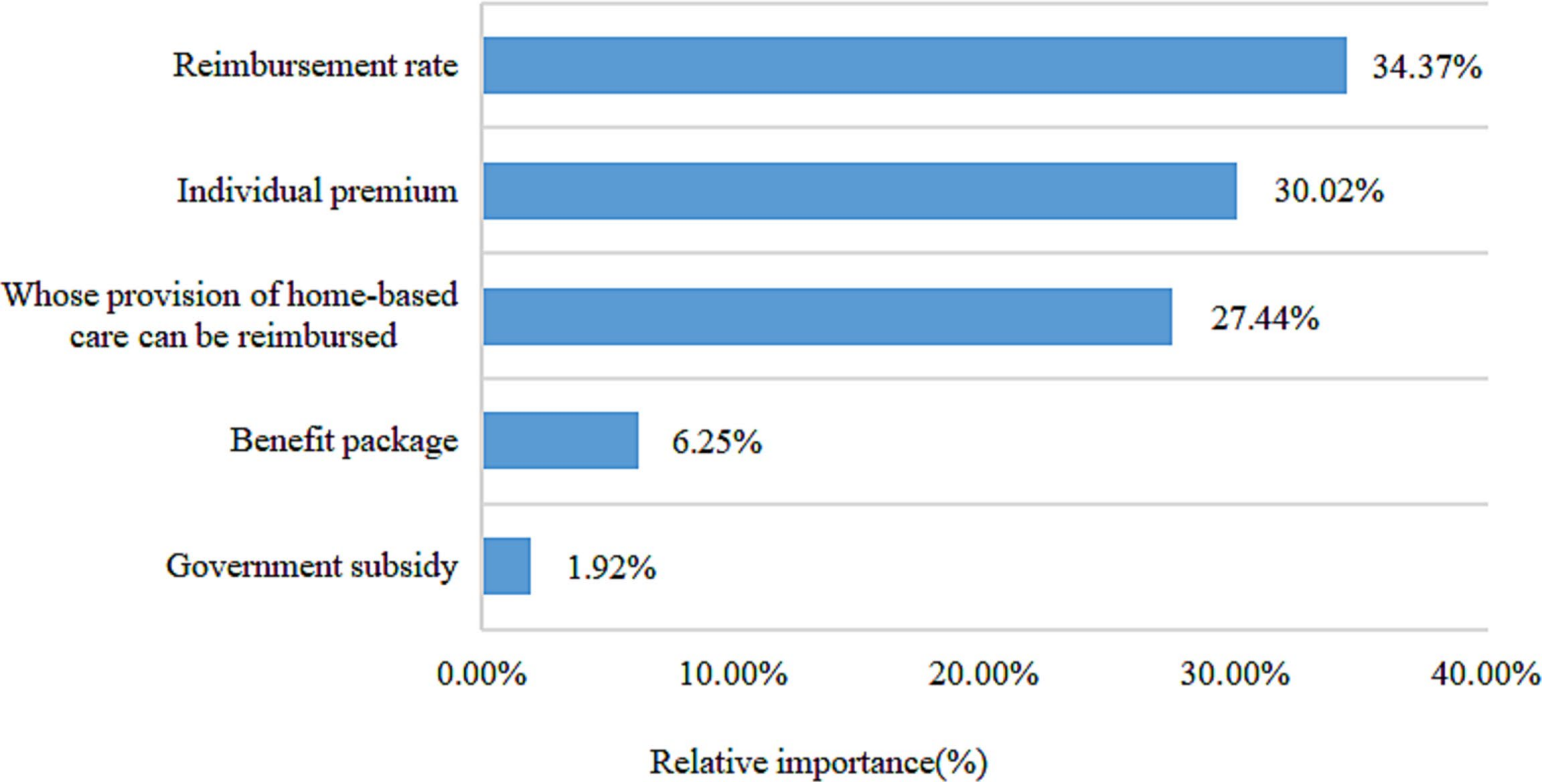
Relative importance of long-term care insurance attributes.

To further calculate the relative importance of each attribute of long-term care insurance, the attribute variables were recoded by effect coding (Table 5). The relative importance of each attribute was calculated by dividing the difference between the lowest and highest coefficients for that attribute by the sum of the differences for all attributes (the coefficient value for the reference level is the negative of the sum of the coefficients for the other levels) (35). As can be seen in Figure 1, the relative importance of the long-term care insurance attributes was ranked as reimbursement rate (34.37%), individual premium (30.02%), whose provision of home-based care can be reimbursed (27.44%), benefit package (6.25%), and government subsidy (1.92%).

**Table 5:** Long-term care insurance preference results based on a mixed logit model (effect coding).

Attributes and levels	Mean	SD
Constant term	−0·232	–
Individual premium		
20RMB/year	Reference	–
70RMB/year	−0.175	–
120RMB/year	−0.487^***^	–
Government subsidy		
0RMB/year	Reference	–
20RMB/year	0.032	0.002
40RMB/year	0.009	0.043
Benefit package		
Medical care + basic life care + rehabilitation training	Reference	–
Medical care + basic life care + rental of assistive devices	−0.117	−0.016
Medical care + basic life care	0.122	−0.424^***^
Reimbursement rate		
65%	Reference	–
75%	0.103	0·562^***^
85%	0.606^***^	1.114^***^
Whose provision of home-based care can be reimbursed		
Professional caregivers only	Reference	–
Professional caregivers and family members, etc.	0.525^***^	0·888^***^
Log likelihood	−1186.586	
Sample size	345	
Observed value	4,140	

SD, standard deviation. ***p < 0.001, **p < 0.01, *p < 0.05.

### 3.4 Subgroup analysis

Taking into account the reality that there are large differences between urban and rural areas in China, we first conducted subgroup analyses by residence type. Preference of participants from the rural areas did not deviate from the results of the full sample, but participants from the urban areas expected government subsidy of 20RMB/year. Additionally, compared with “medical care + basic life care + rental of assistive devices,” “medical care + basic life care +rehabilitation training” was preferred by urban older people (Table 6).

**Table 6:** Regression results of subgroup analysis based on residence type.

Attributes and levels	Rural (*n* = 177)	Urban (*n* = 168)
	Mean	Mean
Constant term	−0·327^***^	−0.670^***^
Individual premium (included in real terms)	−0.0084^***^	−0.008^***^
Government subsidy		
0RMB/year	Reference	Reference
20RMB/year	0.106	0.366^*^
40RMB/year	0.009	0·128
Benefit package		
Medical care + basic life care + rehabilitation training	Reference	Reference
Medical care + basic life care + rental of assistive devices	−0.032	−0.422^**^
Medical care + basic life care	0·149	0·126
Reimbursement rate		
65%	Reference	Reference
75%	0.431^**^	1.281^***^
85%	1.086^***^	1.277^***^
Whose provision of home-based care can be reimbursed		
Professional caregivers only	Reference	Reference
Professional caregivers and family members, etc.	0·823^***^	0·812^***^

***p < 0.001, **p < 0.01, *p < 0.05.

Subsequently, referring to prior study (24), we conducted subgroup analyses based on number of children, chronic conditions, gender, education level and financial situation.

As shown in Table 7, the choice preferences of respondents with two or more children were identical to those of the full sample. There was consistency between the two subgroups in their preferences for the attributes of individual premium, benefit package, reimbursement rate, and whose provision of home-based care can be reimbursed, but there were some differences between the two groups for the attribute of government subsidy. Older adults with one children or without children preferred government subsidy of 40RMB/year, while older people with more children did not have a significant preference for this attribute. Statistical results in Table 8 shows that older people with chronic illnesses considered “basic life care +medical care”sufficient to meet their needs compared with “basic life care+medical care+rehabilitation training.”There was no difference in the statistical results for the gender subgroups compared to the full sample (Table 9), while subgroup analyses based on education level and financial situation yielded meaningful findings (Tables 10, 11). Individual premium lost statistical significance among the higher quality groups (educated, good financial situation). Moreover, government subsidy was preferred by participants who were in average or poor financial shape.

**Table 7:** Regression results of subgroup analysis based on number of children.

Attributes and levels	Number of children≤1 (*n* = 69)	Number of children≥2 (*n* = 276)
	Mean	Mean
Constant term	−0.976^**^	−0.317^**^
Individual premium (included in real terms)	−0.008^**^	−0.010^***^
Government subsidy		
0RMB/year	Reference	Reference
20RMB/year	0.348	0.118
40RMB/year	0.483^*^	−0.036
Benefit package		
Medical care + basic life care + rehabilitation training	Reference	Reference
Medical care + basic life care + rental of assistive devices	0.033	−0·164
Medical care + basic life care	0.561	0.135
Reimbursement rate		
65%	Reference	Reference
75%	1.557^***^	0.465^***^
85%	1.829^***^	0·944^***^
Whose provision of home-based care can be reimbursed		
Professional caregivers only	Reference	Reference
Professional caregivers and family members, etc.	0.594^**^	0.860^***^

***p < 0.001, **p < 0.01, *p < 0.05.

**Table 8:** Regression results of subgroup analysis based on chronic conditions.

Attributes and levels	With chronic diseases (*n* = 229)	Without chronic diseases (*n* = 116)
	Mean	Mean
Constant term	−0.413^***^	−0.418^*^
Individual premium (included in real terms)	−0.010^***^	−0.006^**^
Government subsidy		
0RMB/year	Reference	Reference
20RMB/year	0.106	0·233
40RMB/year	−0.014	0.125
Benefit package		
Medical care + basic life care + rehabilitation training	Reference	Reference
Medical care + basic life care + rental of assistive devices	−0.189	−0.080
Medical care + basic life care	0·264^*^	−0.009
Reimbursement rate		
65%	Reference	Reference
75%	0.716^***^	0.645^**^
85%	1.117^***^	1.181^***^
Whose provision of home-based care can be reimbursed		
Professional caregivers only	Reference	Reference
Professional caregivers and family members, etc.	0.648^***^	1.138^***^

***p < 0.001, **p < 0.01, *p < 0.05.

**Table 9:** Regression results of subgroup analysis based on gender.

Attributes and levels	Female (*n* = 181)	Male (*n* = 164)
	Mean	Mean
Constant term	−0.413^***^	−0.448^**^
Individual premium (included in real terms)	−0.010^***^	−0.007^***^
Government subsidy		
0RMB/year	Reference	Reference
20RMB/year	0·237	0.081
40RMB/year	−0.010	0.073
Benefit package		
Medical care + basic life care + rehabilitation training	Reference	Reference
Medical care + basic life care + rental of assistive devices	−0.167	−0.171
Medical care + basic life care	0.178	0·191
Reimbursement rate		
65%	Reference	Reference
75%	0.628^***^	0·748^***^
85%	1.027^***^	1.200^***^
Whose provision of home-based care can be reimbursed		
Professional caregivers only	Reference	Reference
Professional caregivers and family members, etc.	0.812^***^	0.795^***^

***p < 0.001, **p < 0.01, *p < 0.05.

**Table 10:** Regression results of subgroup analysis based on education level.

Attributes and levels	Uneducated (*n* = 225)	Educated (*n* = 120)
	Mean	Mean
Constant term	−0.549^***^	−0·196
Individual premium (included in real terms)	−0.016^***^	0.001
Government subsidy		
0RMB/year	Reference	Reference
20RMB/year	0·202	0.083
40RMB/year	−0.019	−0.007
Benefit package		
Medical care + basic life care + rehabilitation training	Reference	Reference
Medical care + basic life care + rental of assistive devices	−0.237	−0.067
Medical care + basic life care	0.291^*^	0.009
Reimbursement rate		
65%	Reference	Reference
75%	0.664^***^	0.754^***^
85%	1.028^***^	1.408^***^
Whose provision of home-based care can be reimbursed		
Professional caregivers only	Reference	Reference
Professional caregivers and family members, etc.	0.959^***^	0.732^***^

***p < 0.001, **p < 0.01, *p < 0.05.

**Table 11:** Regression results of subgroup analysis based on financial situation.

Attributes and levels	Average or poor (*n* = 230)	Good (*n* = 115)
	Mean	Mean
Constant term	−0.617^***^	−0.195
Individual premium (included in real terms)	−0.014^***^	0.001
Government subsidy		
0RMB/year	Reference	Reference
20RMB/year	0·265^*^	−0.005
40RMB/year	0.073	−0.100
Benefit package		
Medical care + basic life care + rehabilitation training	Reference	Reference
Medical care + basic life care + rental of assistive devices	−0.177	−0.107
Medical care + basic life care	0.350^**^	−0.043
Reimbursement rate		
65%	Reference	Reference
75%	0·583^***^	1.126^***^
85%	0·842^***^	1.957^***^
Whose provision of home-based care can be reimbursed		
Professional caregivers only	Reference	Reference
Professional caregivers and family members, etc.	1.011^***^	0·527^**^

***p < 0.001, **p < 0.01, *p < 0.05.

## 4 Discussion

This study measured the preference of urban and rural older people in Shandong Province for long-term care insurance using a discrete choice experiment, and analysed their preference, willingness to pay and preference heterogeneity by constructing a mixed logit model. The results of the study show that in terms of preferences for long-term care insurance attributes, older people in Shandong Province preferred lower individual premium, higher reimbursement rate. Compared to only reimbursing home-based care provided by specialists, there was a greater expectation that home-based care provided by professional caregivers and family members, relatives, neighbours, etc. will all be reimbursed.

Consistent with one DCE study of private health insurance (31), in our study, reimbursement rate was the most decisive attribute that older people considered when choosing long-term care insurance. We found that older people had a high willingness to pay for an increase in reimbursement rate. They were willing to pay an additional 128.429 RMB to increase the reimbursement rate from 65 to 85%. It has been similarly found that when co-payment rate is low (i.e., reimbursement rate is high), it leads to a high willingness to pay for some social demographics (16). Reimbursement rate is directly related to the expected benefits that older people receive from long-term care insurance. According to Guo et al.’s projections, the cost of long-term care for the disabled older adults in China will be 5927.5505 billion RMB in 2050 with the high scenario estimates (36), which means that if the long-term care costs are shared to each disabled family, it will bring a heavy financial burden to the family. Therefore, if the level of benefit is low, long-term care insurance has a very limited role in sharing the care burden of family. At present, there is still a large gap between the level of benefits of long-term care insurance for residents and employees in Shandong Province. In Qingdao, Shandong Province, for example, the reimbursement rate for employee LTCI is as high as 90%, while the reimbursement rate for residents participating in the second tier of contributions is only 75% (37). Participants in the resident LTCI scheme are vulnerable to poverty in the event of incapacity as they do not have stable incomes. Therefore, the reimbursement rate of resident long-term care insurance should be appropriately increased on the premise of adhering to the principle of appropriate protection. Higher reimbursement ratio is not only necessary to alleviate the financial burden of disabled families, but also helps to promote equality of access to services for older people at different economic levels (38). In order to raise the level of benefits without increasing the pressure on the payment of the long-term care insurance fund, the target of benefits can be set at older people with severe incapacity and dementia, so as to increase the room for upgrading the level of benefits by narrowing down the scope of the target of benefits (39). In addition, the differentiation of the level of benefits can be set to increase the compensation for older people using home care, which not only complies with the wishes of older people to age at home, but also helps to reduce the cost of long-term care and reduce the pressure on institutional care services (40).

Individual premium was the second most important long-term care insurance attribute. Different countries that have used discrete choice experiments to study preferences for LTCI have almost universally included premiums (16–19), which further confirmed the importance of this attribute. This paper found that older people had a greater preference for long-term care insurance with lower individual premium, which reflects the principle of utility maximization. The price of the insurance product is a significant factor influencing the purchase of long term care insurance (41). Costly premiums can lead to a reluctance to purchase long term care insurance (42, 43). This is in line with the supply and demand equilibrium theory of economics, which states that there is a negative correlation between demand and price when supply is constant (44). By combing through the contribution policies of the pilot cities of LTCI in Shandong Province, we found that at present, the financing of LTCI in most of the pilot cities in Shandong Province mainly came from medical insurance fund and government subsidies, and although some cities have stipulated the responsibility of individual contributions, the actual financing is still transferred from the medical insurance fund, which means that individuals are not making actual contributions to LTCI. Through interviews with staff of the Qingdao Municipal Health Insurance Bureau, we learned that individuals, especially those in rural areas, had a low willingness to contribute to LTCI and raising funds from individuals is difficult. However, individual contribution is an important financing channel for long-term care insurance and relying on medical insurance funds is not sustainable, nor is it consistent with the independence of the insurance design (45). So how to resolve the tension between the importance of individual contributions and the individual’s preference for low premiums? The results of the subgroup analyses may provide meaningful insights. We found that individual premium lost statistical significance among those with higher levels of education and better financial situation. Previous studies have proven that people with higher levels of education (46) and better financial situation (46, 47) tend to show a demand for LTCI. People who are well educated are usually more risk-averse, have a more in-depth knowledge of insurance, and are therefore more willing to contribute to LTCI (48, 49). Better financial situation means greater purchasing power. It is easy to understand that when people’s ability and willingness to contribute increase, they will be less sensitive to premium. Thus, some implications for policy-making can be drawn based on this result to promote the financing of LTCI. First, people’s income level should be raised to improve their purchasing power, and LTCI policy publicity should be strengthened to enhance people’s awareness of LTCI, thus increasing individuals’ willingness to contribute. In addition, it is worth noting that due to the lack of a stable source of income, there is a gap between the contribution ability of participants in the resident LTCI and that of the employee LTCI. In order to ensure the smooth implementation of the resident LTCI, a transfer fund system can be established with reference to Qingdao’s practice. The transfer between the employee and resident long term care funds will make up for the shortage of the resident fund. Finally, from the perspective of integration, unifying employee long-term care insurance and resident long-term care insurance can achieve risk sharing on a broader scale, which not only helps to reduce the pressure on individual contributions, but also solves the problem of fragmentation of the system (50).

Long-term care insurance, which reimburses home-based care provided by both professionals and family members, can be more effective for older people than long-term care insurance, which reimburses only home-based care services provided by professionals. This means informal care services were still favored by older people. Consistent with the findings of this paper, a large number of studies from China have demonstrated that the majority of older people would prefer to have family members provide long-term care for them (51, 52). The reasons can be explained in two ways: first, the traditional Chinese culture of filial piety and the concept of “bring up children for the purpose of being looked after in old age” have a strong influence. Second, there is greater familiarity and emotional ties between family members and older people, being cared for by someone familiar is more in line with the habits and psychological needs of older people (53, 54). It is worth noting that previous studies have found that older people who are women, more educated, in a better financial situation, and with fewer children are more likely to choose professional caregivers to provide long-term care for them (55–58). However, the subgroup analyses in our paper did not find differences in preferences for long-term care providers among older adults with different socioeconomic characteristics. All subgroups had significant preferences for informal caregivers. This enlightens us that we should advocate and support families to play a greater role in long-term care (59). From the perspective of policy design, compared with the long-term care insurance policy that only supports formal care, the long-term care insurance policy that supports both formal care and family care has a crowding-in effect on family care (60). Therefore, support for informal caregivers such as provision of cash benefits should be added to the long-term care insurance policy, which not only offsets the opportunity cost of caring for the older adults to a certain extent, thus encouraging informal caregivers to take the initiative to assume the responsibility of caregiving (60, 61), but also conforms to the international trend that responsibility for long-term care is gradually returning from the state to individuals and families (62). The professionalism of informal careers can be enhanced through regular professional training (63). Meanwhile, it is important to note that certain long-term care services are too specialized to be accomplished by family members alone, therefore, the intervention of professional caregivers is essential and formal and informal care should be integrated in the long run.

Government subsidy and richer benefit packages are often thought to increase the people’s willingness to enroll in LTCI (26). In this study, these two attributes lost statistical significance in the mixed logit model with the full sample. However, the mixed logit model for the full sample only told part of the story, and subgroup analyses were able to paint a more nuanced picture of the heterogeneity of respondents’ preferences. We found some meaningful insights in the subgroup analysis.

Firstly, older people who were in average or poor financial situation and had fewer children were found to have a significant preference for government subsidy. Government subsidy can increase residents’ disposable income by lowering the price of LTCI, thus triggering an increase in demand for LTCI (64). However, due to people have different price elasticity coefficients, the same government subsidy will have different impacts on different residents, and the government subsidy will produce a more pronounced utility enhancement for residents with low incomes (65), which may explain why government subsidy had an impact on older people in average/poor financial situation, while they had no impact on older people in good financial situation. The fewer children there are, the less support the older adults will receive from their children (66), so older people with fewer children hoped to receive external support from government. Secondly, people with chronic diseases are assumed to prefer more generous long-term care because they are more likely to use long-term care services in the future. However, contrary to this hypothesis, our paper found older people suffering from chronic diseases showed preference for basic long-term care services (medical care + basic life care) rather than expanded benefit package (medical care + basic life care+rehabilitation training). While this group has high long-term care needs, they may have limited knowledge of the content of long-term care services, and thus were satisfied with basic benefit packages. Finally, there are also significant differences in the preferences for government subsidy and benefit package between urban and rural older people. Urban older people had a clear preference for benefit package including “medical care + basic life care + rehabilitation training,” while benefit package was not a key factor that rural older people considered when choosing LTCI. This may be due to the scarcity of avaliable care services in rural areas (67), resulting in limited awareness of long-term care services among rural older people. Compared to rural older people, urban older people preferred government subsidy. This is because the problem of low birth rates and smaller family structures in urban areas is more severe (66), and the limited family resources available to urban older people require government support. In general, the above discussion provides several policy implications. On the one hand, financial subsidy should be rationally set according to the actual needs of different residents (64), and older people with lower income and fewer children are the key target group for government subsidy. On the other hand, long-term care knowledge should be popularized, especially in rural areas, to enhance older people’s understanding of long-term care, thus helping them to have a more scientific understanding of their own needs.

There are some limitations to this study: first, long-term care insurance decision-making is a complex process that is influenced by many factors. Only five long-term care insurance attributes were included in this study, future research should further explore the impact of other potential attributes on long-term care insurance selection preferences. Second, due to the limitations of human and material resources, this study was conducted only in the scope of Shandong Province, and although it met the research needs, the generalizability of findings to other areas in China is limited, therefore, the research sample should be further expanded in the future so as to come up with more comprehensive policy recommendations. Third, discrete choice experiment measures stated preference based on hypothetical scenarios, which is susceptible to hypothetical bias, and the results may differ from the decisions people make when faced with actual choices, further research should be conducted on older people’s revealed preferences for LTCI to verify the findings of this paper. Fourth, we used cross-sectional data without considering whether there is inconsistency in the preferences of older adults over time, this should be improved in future research. Finally, due to limitations in mobility, some vulnerable groups such as severely disabled older people may not have been included in our survey, potential sample selection biases may have an impact on our research results. Stricter sampling procedures should be designed to overcome sample selection bias.

## 5 Conclusion

This paper investigated the preferences of urban and rural older people in Shandong Province for long-term care insurance based on a stated preference approach, and found that individual premium, reimbursement rate, and whose provision of home-based care can be reimbursed had an impact on older people’s choice of long-term care insurance. Older people with different characteristics had different preferences for individual premium, government subsidy and benefit package. Improving policy design of LTCI according to the preferences of the target population is essential for the sustainability of long-term care insurance.

## Data Availability

The raw data supporting the conclusions of this article will be made available by the authors, without undue reservation.
